# Illness experience and coping styles of young and middle-aged patients with sudden sensorineural hearing loss: a qualitative study

**DOI:** 10.1186/s12913-021-06763-z

**Published:** 2021-07-27

**Authors:** Yang Yuan, Hong Wang, Qiuyun Chen, Congyan Xie, Haixia Li, Lu Lin, Li Tian

**Affiliations:** 1 The First Affiliated Hospital of Soochow University/School of Nursing, Medical College of Soochow University, No. 188 Shizi Road, 215006 Suzhou, People’s Republic of China; 2 School of Nursing, Medical College of Soochow University, 215006 Suzhou, China; 3 Suzhou Kowloon Hospital, Shanghai Jiao Tong University School of Medicine, 215021 Suzhou, China

**Keywords:** Sudden sensorineural hearing loss, Young and middle-aged, Illness experience, Coping style

## Abstract

**Background:**

The incidence of Sudden Sensorineural Hearing Loss (SSNHL) is increasing and tends to occur at a young age. The patient’s disease experience during treatment is related to their physical and mental health. Effective coping styles such as proactively solving problems and asking for help will alleviate the patients’ psychological symptoms and improve their quality of life.

**Aims and objectives:**

To explore the illness experience and coping styles of young and middle-aged patients with SSNHL (age: 18–64 years), clarify the relationship between the two, and understand the psychological state and needs of the patients.

**Methods:**

The purposive and maximum difference sampling method was used to conduct semi-structured interviews with 23 young and middle-aged SSNHL patients. The interview data were analyzed by Colaizzi’s seven-step analysis.

**Results:**

The illness experience of young and middle-aged SSNHL patients was complex, including symptoms during the onset of deafness and emotional experience before and after diagnosis. The coping styles of young and middle-aged SSNHL patients were active and diverse, including active acquisition of information, change in living habits, and seeking the care and attention of medical staff. Illness experience and coping style influence each other: good illness experience leads to active coping styles; active coping style results in good illness experience.

**Conclusions:**

The illness experience of young and middle-aged SSNHL patients includes not only physical symptoms, but also changes in psychological and emotional reactions. Good illness experience can lead patients to adopt active coping style. Active and effective coping styles, such as positive acquisition of information, change in living habits and seeking care and help, can improve patients’ illness experience.

**Supplementary Information:**

The online version contains supplementary material available at 10.1186/s12913-021-06763-z.

## Introduction

Sudden Sensorineural Hearing Loss (SSNHL) refers to the sensorineural hearing loss of unknown cause that occurs suddenly at 72 h, and the hearing loss is ≥ 30 dBHL on at least 3 frequencies [1]. The annual incidence rate in the United States is 5–27 per 100 thousand people, and the annual incidence rate in China is 2–20 per 100 thousand people [2]. SSNHL can occur at any age, and the incidence is increasing and there is a trend for it to occur at younger ages [3]. Three epidemiological surveys in Japan have found that the incidence of SSNHL is increasing year by year, which is 3.9 per 100 thousand people (1972), 14.2 per 100 thousand people (1987), 19.4 per 100 thousand people (1993), and 27.5 per 100 thousand people (2001) [3]. Therefore, it is important to pay attention to the quality of life of SSNHL patients, especially young patients. Illness experience includes not only changes in physical symptoms, but also changes in psychological feelings. The patient’s illness experience throughout the treatment process will affect their cognition and attitude towards the disease, and in turn affect their mental and psychological state [4]. SSNHL is often accompanied by many uncomfortable symptoms such as tinnitus, dizziness, hearing, and vision loss [3], and patients are prone to mental and psychological disorders such as anxiety and fear [5], which significantly affect their daily life, work, and social interaction. As the mainstay of family and society, young and middle-aged SSNHL patients are more likely to feel various types of pressure due to the impact of disease on life or work, which makes the incidence of anxiety and depression in young and middle-aged SSNHL patients significantly higher than other groups. The illness experience can be conceptualized as a psychopathological phenomenon, which should be regarded as a general symptom of the disease and should be recognized and processed [6]. Coping style is the way people consciously adjust their cognition and behavior when facing stress, and it is an important intermediate mechanism between stress and health, which has a significant impact on the physical and mental health of patients [7]. When patients suffer from stressful events, they will instinctively adopt certain coping measures, and different coping styles have different effects on their stress levels. If the coping style is effective, the patient’s psychological symptoms will be alleviated; otherwise, if the coping style adopted is inappropriate, it can easily develop into acute stress disorder (ASD) or even posttraumatic stress disorder (PTSD) [8]. In addition, coping styles play a key role in predicting the patient’s supportive experience during and after illness [9], which helps medical staff to identify patient needs and provide timely support to patients. It can be seen that strengthening the evaluation of patients’ illness experience and helping patients to establish effective coping style is very important in the management of SSNHL patients. Therefore, this study aimed to interview patients during their hospitalization and use qualitative research methods to analyze patients’ illness experience and coping styles after the onset of SSNHL in order to gain an insight into the coping styles of SSNHL patients in the hope of improving their illness experience.

## Methods

This study was performed following the Consolidated Criteria for Reporting Qualitative Research (COREQ) checklist [10] and acquired ethical approval from the Medical Ethics Committee of Soochow University (No. SUDA 20200515H02). All participants signed the informed consent form.

### Design

This study used the maximum difference sampling method to recruit research participants in order to make the sample representative. In-depth semi-structured interviews were used to collect data, aiming to explore the illness experience and coping styles of young and middle-aged SSNHL patients and understand their psychological state and needs so as to provide reference for the development of clinical nursing interventions for patients’ coping styles that can improve their illness experience. The interview data were analyzed through Colaizzi’s seven-step phenomenological analysis, including: (i) carefully read all interview records; (ii) extract important statements; (iii) code recurring and meaningful opinions; (iv) collect the coded opinions; (v) write a detailed description without omission; (vi) identify similar views and summarize themes; (vii) return to the interviewee for verification [11] and the NVivo 11 software was used for data management.

### Participants

In this study, SSNHL patients who were willing to be interviewed were selected from inpatients in the Department of Otolaryngology in a level A tertiary hospital (having more than 500 beds and representing a advanced medical level according to the Hospital Grading System in China) in Suzhou, China from December 2019 to March 2021. Purposive and maximum difference sampling method was used to recruit SSNHL patients who could provide the most abundant information. The recruitment of patients was mainly based on the degree of hearing impairment, age, gender, and educational level of the patient. The degree of hearing impairment was based on the results of pure tone audiometry (PTA) in patients’ medical records. The determination of the sample size was bounded by the saturation of the data needed for the research, that is, no new information emerges from the new data. Inclusion criteria for patients: (i) hospitalized patients who met the diagnostic criteria of SSNHL in the 2015 SSNHL Diagnostic and Treatment Guidelines; (ii) young and middle-aged (18 years ≤ Age ≤ 64 years); (iii) have basic reading comprehension and audiovisual expression skills; (iv) volunteer to participate in this research.

### Establishing the interview outline

According to the purpose of research and based on relevant literature, interview questions were designed. In November 2019, three participants were recruited for piloting. Based on the results of piloting data analysis and the recommendations of otolaryngology and psychology experts, the interview outline was revised and finalized (See Additional file 1). The interview outline was not static and could be flexibly adjusted according to the patient’s specific answers, mainly about the patient’s physical and mental experience, changes in behavior, and psychological needs after illness.

### Data collection and analysis

The one-on-one in-depth interviews were used for data collection. Before the start of each interview, the researcher explained the purpose of the research to the interviewee, promised to protect the privacy and security of the interviewee, and signed an informed consent form after obtaining the consent of the interviewee. Each interview lasted for 30–40 min. When the interviewee was available or in remission of SSNHL disease, a quiet, comfortable, and relatively independent space was chosen to conduct the interview based on the outline. Appropriate communication skills were used, such as listening, empathy, repetition, clarification, and reaction in order to reflect the patients’ opinions and attitudes as accurately and comprehensively as possible. The interview was recorded with a voice recorder, and the non-verbal behaviors of the interviewee were observed and documented in words in detail during the interview.

The transcription of data followed the principles of timeliness, uniform format, verbatim record, and double backup. The collation and analysis of data were carried out at the same time. After each interview, the text transcription was started, and the patient’s pitch and pauses during the conversation were documented in words in detail. The transcription was carried out by two uniformly trained researchers, one of whom was an interviewer or recorder. One researcher first transcribed the recording and then the other reviewed the transcript. The transcript was returned to the interviewee for verification before analysis, and the controversial content was verified and modified in time to ensure the accuracy of the data. Using Colaizzi phenomenological data analysis method [11], the interview records were carefully read. Meaningful statements were extracted and coded, and recurring views were classified using NVivo 11 software. A detailed, exhaustive description was written; similar points of view were identified; the theme was refined.

### Ethical considerations

Ethical approval to perform the study was obtained from the Ethics Committee of Soochow University (Ethical Approval No. SUDA20200515H02). Each interviewee signed an informed consent form prior to the interview and registered their general demographic data. During the interview, interviewees may withdraw from the interview at any time without any reason, which did not happen in this study.

## Results

A total of 23 patients were interviewed, numbered P1-P23. All participants currently live in Suzhou, China. The average age was 35.26 years old (range: 18–53 years), including 12 males and 11 females. Table 1 provides further details about interview participants.

**Table. 1:** General information of the interviewees (*n* = 23)

Interviewee	Gender	Age (years)	Occupation	Education	Marital status	Affected ear	Hearing in the affected ear during admission(dB)	Hearing in the affected ear during interview(dB)
P1	Male	40	Businessman	Junior college	Married	Left	L: 70	L: 70
P2	Male	28	Business manager	University/college	Unmarried	Left	L: 70	L: 50
P3	Male	50	Planner	University/college	Married	Right	R: 80	R: 70
P4	Female	20	Kindergarten teacher	University/college	Unmarried	Binaural	L: 30 R: 40	L: 30 R: 40
P5	Female	48	Doctor	Master’s degree	Married	Left	L: 100	L: 100
P6	Male	23	Staff	Technical secondary school	Married	Binaural	L: 30 R: 25	L: 25 R: 25
P7	Male	45	Engineer	University/college	Married	Right	R: 45	R: 30
P8	Female	32	Drug researcher	Doctoral degree	Married	Left	L: 40	L: 30
P9	Female	33	Deputy store manager	University/college	Unmarried	Binaural	L: 40 R: 40	L: 30 R: 30
P10	Female	43	Housewife	High school	Married	Binaural	L: 40 R: 40	L: 40 R: 40
P11	Male	18	Student	High school	Unmarried	Binaural	L: 50 R: 55	L: 40 R: 45
P12	Male	37	Semiconductor R&D	University/college	Married	Right	R: 45	R: 35
P13	Female	27	Financial sector	Master’s degree	Unmarried	Left	L: 30	L: 25
P14	Female	30	Doctor	University/college	Married	Left	L: 35	L: 30
P15	Male	53	Corporate	University/college	Married	Binaural	L: 65 R: 50	L: 50 R: 45
P16	Male	33	Nurse	University/college	Married	Binaural	L: 50 R: 60	L: 50 R: 60
P17	Female	38	Kindergarten teacher	Junior college	Married	Left	L: 45	L: 40
P18	Male	30	Tax officer	Master’s degree	Married	Right	R: 40	R: 35
P19	Female	20	Student	University/college	Unmarried	Left	L: 45	L: 40
P20	Male	33	Businessman	University/college	Unmarried	Left	L: 70	L: 60
P21	Male	32	Production manager	University/college	Married	Left	L: 100	L: 80
P22	Female	46	Staff	University/college	Unmarried	Right	R: 40	R: 40
P23	Female	52	Cleaner	Primary school	Married	Left	L: 60	L: 60

Notes: *P* Patient, *L* Left, *R* Right, *R&D* Research and Development

### Overview

**Fig. 1:**
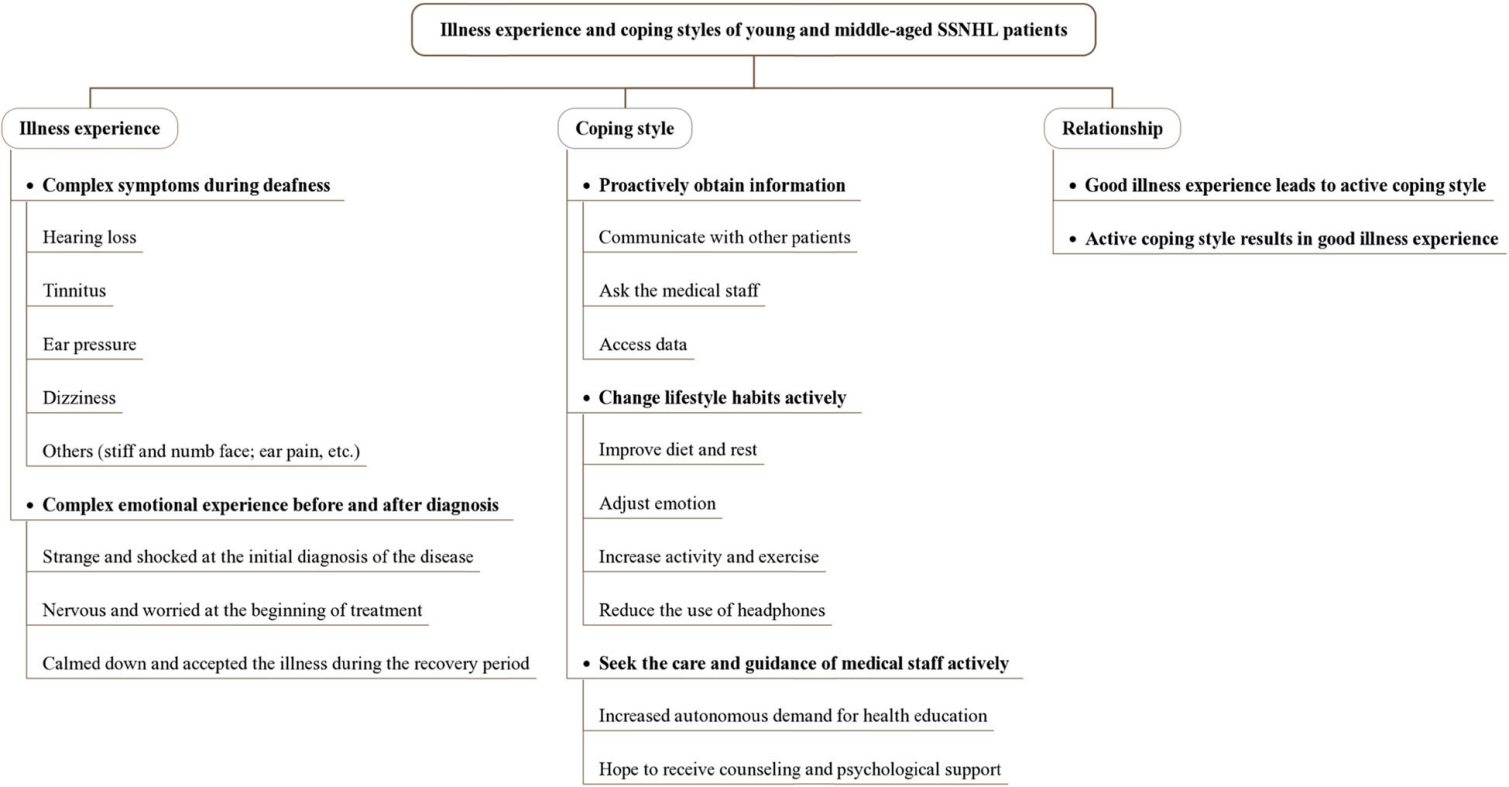
Overview of the main themes and sub-themes

The interview data generated three main themes: (i) The illness experience of young and middle-aged SSNHL patients was complex: complex symptoms during the onset of deafness and complex emotional experience before and after diagnosis. (ii) The coping styles of young and middle-aged SSNHL patients were active and diverse: active acquisition of information, change in living habits and seeking the care and attention of medical staff. (iii) Illness experience and coping style influence each other: good illness experience leads to active coping styles; active coping style results in good illness experience. The generated themes and sub-themes are visualized in Fig. 1.

### Illness experience

#### Complex symptoms during deafness

The majority of patients suffered from tinnitus symptoms, and 20 patients mentioned that they had tinnitus when they developed symptoms. Seven patients had symptoms of hearing loss immediately upon onset. Fourteen patients (61 %) had symptoms of pressure in the ear cavity and dizziness. Only three patients had other symptoms such as facial stiffness, numbness, or ear pain, etc. Complex symptoms and feelings brought many inconveniences to patients’ life or work, which in turn worsened the illness experience of some patients.

“I felt the ‘buzzing’ in my ears and I couldn’t hear the sound. This affected my normal communication with others at work, making me very distressed. ” (P3).

“Suddenly there was a tinnitus sound at night, similar to the ‘boom, boom’ sound of flushing the toilet, making me unable to sleep peacefully.” (P12).

#### Complex emotional experience before and after diagnosis

##### Strange and shocked at the initial diagnosis of the disease

Some patients had never heard of the disease or did not understand the disease. Therefore, when they first learned about the diagnosis of SSNHL, they were surprised. And some patients were shocked that deafness would happen to them, saying that it was unacceptable.

“As an otolaryngologist, I was in a bad mood after being diagnosed at that time, thinking about how the disease fell on me.” (P5).

“At the time I thought this diagnosis was a bit strange. After all, my hearing didn’t drop too much, I just felt that my tinnitus was a bit uncomfortable.” (P6).

##### Nervous and worried at the beginning of treatment

After the diagnosis, 14 patients were worried about the recovery from and prognosis of the disease, and they experienced nervous and anxious emotions.

“I was very nervous at first because I was still young and worried that it would affect my future development.” (P1).

“Because I heard the word ‘deaf’ at the time, I was worried about ‘hearing losses. I suddenly became nervous and worried whether I would be ‘deaf’.” (P13).

##### Calmed down and accepted the illness during the recovery period

Although the emotional experience of patients in the early stages of illness was mostly nervous and anxious, most of the patients’ emotions tended to calm down after a period of treatment and recovery. Fifteen patients mentioned that they could actively participate in treatment with a calm mind.

“After several days of treatment, my hearing feels better and I am confident that it can be cured.” (P7).

“I feel more at ease in the hospital now. I think there is still hope and active treatment is needed.” (P8).

### Coping style

#### Proactively obtain information

##### Communicate with other patients

Patients obtained more disease-related information from patients in the same hospital or other relatives and friends who had the same disease and similar experience.

“During hyperbaric oxygen therapy, I came into contact with some sudden-deaf patients and learned some disease information of different reliability.” (P2).

“A colleague told me that he also had ‘tinnitus’ and the disease should be treated quickly.” (P7).

##### Ask the medical staff

Seven patients could use the resources around them. They consulted doctors, nurses, or classmates and friends who were engaged in medical work.

“After I was hospitalized, I asked some of my classmates who were medical majors and the chief physician to learn about the disease.” (P8).

“My mother (who used to be a nurse) found out some causes of the disease.” (P11).

##### Access data

Due to lack of knowledge about SSNHL after diagnosis, the patient chose to take the initiative to look up information about the disease through the Internet or literature. But some information on the Internet may lack scientificity.

“I also did some literature search on my own, such as websites like The Lancet.” (P2).

“I have looked up some relevant information online.” (P18).

“Although I have checked the information on the Internet, I feel that what is said online is not always correct.” (P22).

#### Change lifestyle habits actively

##### Improve diet and rest

In the interview, 18 patients stated that they tried to improve their previously unhealthy eating habits after illness. Thirteen patients mentioned that they should pay more attention to their sleep quality and daily routines.

“The doctor told me to limit the intake of coffee and chocolate as much as possible. In fact, I like having them pretty much, but in order to recover from the disease, I will refrain.” (P4).

“I need to pay attention to my poor sleep quality and try to improve it.” (P7).

“Now I am having a basically a low-salt and low-fat diet.” (P14).

##### Adjust emotion

The patient mentioned that their illness might be related to emotional instability and irritability. Therefore, 13 patients paid more attention to adjusting their mental state after diagnosis.

“It is said on the Internet that irritability is not good for recovery from the disease, and I recently started to adjust my state.” (P6).

“Now I will tell myself to try my best to keep a peaceful mind and don’t worry.” (P8).

“I try to adjust my mood and relax myself.” (P19).

##### Increase activity and exercise

Twelve patients realized the importance of relieving pressure in life and work, doing physical exercise in a timely manner and at a reasonable intensity after illness.

“I will limit the time spent doing such (strenuous) sports.” (P11).

“I rarely exercise when I am home after work. I will increase my exercise time reasonably in the future.” (P12).

##### Reduce the use of headphones

Some patients used headphones quite frequently before the onset. 16 patients mentioned that they would pay attention to reducing the frequency of using headphones.

“I sometimes use headphones. Now I will try my best to control the time of use.” (P12).

“I used earphones a lot before I was hospitalized. From now on I will refrain from using headphones as much as possible.” (P19).

#### Seek care and guidance from medical staff actively

##### Increased autonomous demand for health education

More than half of the patients (16) hoped that medical staff could provide health education about medication and disease-related knowledge.

“I hope that medical staff will explain to patients the probability, time and cost of recovery with treatment.” (P2).

“I want to know about drug contraindications and side effects of drugs. Once it occurs, I know how to deal with it.” (P3).

“The education of disease knowledge is very important. I hope that medical staff can make some popular science leaflets in the form of QR codes and WeChat applets.” (P13).

##### Hope to receive counseling and psychological support

From onset to diagnosis, and then to hospitalization, the patient experienced a complex psychological process such as shock, nervousness, fear, anxiety, etc. The 18 patients hoped that the medical staff could provide counseling and psychological support, care for them and help them face difficulties together.

“Sometimes I hope that doctors and nurses can communicate more from the perspective of the patient, so that it will be easier for the patient to accept the disease psychologically.” (P1).

“I hope that when the doctor comes to do rounds every day, he can pay more attention to the patient’s condition and ask the patient how they feel after treatment.” (P12).

“I hope they can provide more psychological guidance, because patients with this disease will feel a little nervous.” (P13).

### The relationship between illness experience and coping style

#### Good illness experience leads to active coping style

Nine patients were optimistic and calm after diagnosis, and adopted active and effective coping styles for treatment.

“After I noticed hearing loss and tinnitus in my left ear, I hurried to the hospital for treatment. I can be fully engaged in the treatment.” (P2).

“I am not very worried, so I have always been calm. I have looked up some relevant information on the Internet and understand that this treatment may take a long time, but I will stick to the treatment.” (P14).

#### Active coping style results in good illness experience

After a period of treatment, through various positive coping styles, such as improving living habits and actively obtaining disease information, the symptoms of the disease in SSNHL patients were alleviated; the patient’s condition was restored, and their state of mind gradually calmed down.

“After a period of treatment, I slowly began to accept the reality of deafness. I think it is useless to worry. It might be better to adapt to it.” (P1).

“After a few days of treatment, my hearing feels better and I am confident that it can be cured. I have to pay attention to the problem of poor sleep quality. I follow your advice and plan to go to the neurology department after discharge from the hospital, and use some sleep-promoting medications to improve sleep.” (P7).

## Discussion

The onset of SSNHL is sudden, and the patient’s illness experience is complicated. In a short period of time, patients may experience many symptoms such as hearing loss, tinnitus, dizziness, and even facial numbness and stiffness. The patient experienced complex psychological changes from shock to worry to calmness during the disease process. The results of this study showed that patients had relatively positive coping styles, and patients’ illness experience and coping styles influenced each other. Good illness experience can lead patients to adopt active coping style; active and effective coping style can improve patients’ illness experience, such as positive acquisition of information, change in living habits and seeking care and help. In addition, patients needed the help from medical staff (including disease-related information and psychological counseling), therefore medical staff need to identify patients’ illness experience in time, satisfy the reasonable needs of patients, encourage, and help patients to adopt active coping styles to overcome the discomfort experience caused by the disease.

The inducing and influencing factors of SSNHL are diverse [12]. Psychology, drug toxicity, rest and sleep, and noise in the environment may affect the onset of SSNHL symptoms [12]. A qualitative interview of young patients with chronic conditions showed that their illness experience was complex and intense and they needed active support [13], which is consistent with our conclusion. For example, they wanted to be noticed and listened to when expressing their feelings, and they wanted to seek more care instead of being ignored [13]. Our results showed that young and middle-aged SSNHL patients had a variety of symptoms at the onset and wanted more care and attention as well. On the other hand, the results of this study showed that after the onset of disease, patients tried to prevent the aggravation or recurrence of the disease by changing unhealthy lifestyles, for example, improve diet, pay attention to rest, adjust emotions, exercise moderately and reduce the use of headphones. These positive coping styles should be encouraged. However, the interview also revealed some problems: some SSNHL patients lacked disease-related knowledge and sought psychological care. Therefore, helping patients to acquire disease-related knowledge, timely identifying patients’ disease experience, and maintaining patients’ mental health is essential to SSNHL symptom management. Furthermore, the results of this study showed that there was a correlation between the illness experience and coping styles of young and middle-aged SSNHL patients. Positive and good experience guided the patients to respond actively. Patients with optimistic and calm emotional experience could bravely face the disease and seek medical treatment in time, and take a positive approach to working with the medical staff during treatment Positive coping styles brought good experience to patients. In this study, young and middle-aged SSNHL patients adopted various positive coping styles, such as improving their lifestyles and proactively acquiring disease-related information, etc. The patients’ disease symptoms gradually reduced, and their mental state was also improved. What is more, studies have shown that coping styles play a key role in predicting the support experience of patients during and after illness [9], which helps medical staff provide timely support to patients. Therefore, the patient’s illness experience and coping styles are related to each other, and jointly affect the patient’s treatment effect.

A study pointed out that paying close attention to patients’ negative coping styles and educating and training patients to improve their coping skills may help improve their quality of life [14]. According to Patient P22, although they could learn about the disease by communicating with other patients or searching for information online, the quality of the information varied. A study also reported that patients had an urgent need for disease-related knowledge, but lacked the ability to assess the reliability and scientificity of information sources [15]. Therefore, professional health education is very important for SSNHL patients [16]. Medical staff can teach patients to obtain information from scientific sources and improve health education for SSNHL patients [15]. The needs of patients can be investigated, and the disease-related knowledge needed by patients can be emphatically explained according to individual differences [16]. At the same time, medical staff can choose easy-to-understand teaching methods [17], such as using animation and scenario simulation, and provide timely feedback on the health education effects of patients. Patient P13 suggested developing an SSNHL knowledge exchange platform through APPs, WeChat applets, etc. Professional medical staff can do health education and publicity campaigns on the platform, and patients can share treatment experience with each other on the platform, so as to improve patients’ awareness of the disease, prevent recurrence, and facilitate patients’ recovery.

On the other hand, strengthening the psychological support for SSNHL patients was also an important point from the interview. It is necessary to provide patients with psychological counseling during the treatment and recovery period. The patient’s fear of the disease will lower their confidence in treatment, and even compromise their compliance with treatment, thereby affecting their recovery [17]. Shao’s research pointed out that supportive social relationships can reduce the impact of stressors on patients and have an important regulatory effect on their physical and mental health [18]. As many patients mentioned in the interview, they needed psychological support from medical staff. Medical staff need to help patients actively cope with the disease, provide them with emotional support and psychological counseling, and communicate with them effectively to understand their needs and meet their reasonable needs. For patients with moderate to severe deafness, gestures, pictures, and videos can be used [17]. When patients are admitted to the hospital, the medical staff can identify the possible psychological problems of the patients in a timely manner based on understanding of their general information and disease status. As Patient P12 said, paying attention to the interaction with patients during their hospitalization to help resolve their psychological problems, increase their confidence in treatment, reduce their psychological stress, and prevent the occurrence of adverse psychological stress events. Yilmaz suggested that good family support can make patients feel love and dignity, and promote the conversion of their family roles [19]. Medical staff should encourage the patient’s family to care for the patient, and emphasize the role of family members in the patient’s recovery from illness, which can reduce the patient’s psychological burden and mental pressure in varying degrees [18]. Due to work pressure, some young and middle-aged patients cannot be fully engaged in treatment, thus resulting in recurrence of the disease. The patients need the support and help from family members and society, so that they can have enough time and energy to invest in treatment, and thus promoting disease recovery and improving patients’ quality of life [19].

In summary, on the one hand, medical staff can guide patients to obtain effective disease-related information from scientific sources according to the different needs of patients, encourage patients to share treatment experience with each other, improve health education for SSNHL patients, and raise patients’ awareness of the disease. Moreover, medical staff should promptly identify patients’ negative illness experience, help patients actively cope with the disease, and provide patients with emotional support and psychological guidance. On the other hand, promoting family support and social support is of great significance for reducing the physical and mental burden of young and middle-aged SSNHL patients, and improving their illness experience and quality of life.

### Strengths and limitations

One of the strengths of this study was that it identified the main care needs of patients by exploring the illness experience and coping styles of young and middle-aged SSNHL patients as well as the relationship between illness experience and coping styles, and made suggestions for clinical nursing practice to optimize sudden deafness management.

This qualitative study also had some limitations. The selected interviewees were young and middle-aged people, and there was no comprehensive research and analysis for different age groups. Different groups of people have different needs. For example, young patients are more likely to have psychological needs because the disease has a more serious impact on their work; however, for elderly patients, due to the decline in physical function, deafness has a greater impact on their lives [20]. Moreover, limited by the research data, we only discussed the coping styles of the participants after the onset of SSNHL, and could not compare the changes in their coping styles pre-post diagnosis. In addition, this qualitative research recruited patients from one research site only, compromising the representativeness of the sample. In future research, a multi-center, full-cycle, and cross age group design can be adopted.

## Conclusions

The illness experience of young and middle-aged SSNHL patients is complex, including not only physical symptoms, but also changes in psychological and emotional responses. Good illness experience can lead patients to adopt active coping style. Active and effective coping style can improve patients’ illness experience, such as positive acquisition of information, change in living habits and seeking care and help. Timely identification of patients’ illness experience by medical staff combined with psychological counseling may help patients improve their illness experience and quality of life.

## Supplementary Information

**Additional file 1. **Interview Outline

## Data Availability

The datasets used and/or analyzed during the current study available from the corresponding author on reasonable request.

## Abbreviations

SSNHL: Sudden Sensorineural Hearing Loss
ASD: Acute Stress Disorder
PTSD: Posttraumatic Stress Disorder
PTA: Pure Tone Audiometry
