# Supplementary material for: Illness experience and coping styles of young and middle-aged patients with sudden sensorineural hearing loss: a qualitative study
Source: BMC Health Serv Res. 2021 Jul 27;21:742. doi: 10.1186/s12913-021-06763-z (PMC8314487; doi:10.1186/s12913-021-06763-z)
Supplement: Supplementary file 1 — Additional file 1. Interview Outline [file 12913_2021_6763_MOESM1_ESM.docx]

**Appendix: Interview Outline**

(i) Can you describe in detail what happened to your deafness? (Symptoms, feelings, and duration of the attack);

(ii) What factors do you think caused or aggravated these symptoms or feelings?

(iii) How did these symptoms or feelings affect your daily life and work?

(iv) Can you describe your mood when you were just diagnosed with sudden deafness? How are you feeling now? What has changed? Why did this change happen?

(v) What information did you get during the treatment? How to recognize this information?

(vi) How did you deal with the deafness? What adjustments have you made in terms of diet, rest and activity, exercise, mood adjustment, and use of electronic products (including headphones)? Why did you make these adjustments?

(vii) Can you describe in detail the types of medications currently being taken and their effects and precautions respectively? Have you ever missed/stopped taking medication or reduced the dose of medication by yourself? If so, what was the reason? What kind of effect do you think these medication behaviors will have on your condition? What measures have been taken to make up for your medication behaviors?

(viii) What kind of help do you expect from medical staff in managing sudden deafness symptoms?
